# Prognostic significance of CD4-positive regulatory T cells in tumor draining lymph nodes from patients with bladder cancer

**DOI:** 10.1016/j.heliyon.2020.e05556

**Published:** 2020-12-01

**Authors:** Ali Ariafar, Yasmin Vahidi, Maryam Fakhimi, Ardalan Asadollahpour, Nasrollah Erfani, Zahra Faghih

**Affiliations:** a Urology-Oncology Research Center, Shiraz University of Medical Sciences, Shiraz, Iran; b Department of Urology, School of Medicine, Shiraz University of Medical Sciences, Shiraz, Iran; c Shiraz Institute for Cancer Research, School of Medicine, Shiraz University of Medical Sciences, Shiraz, Iran; d Department of Immunology, School of Medicine, Shiraz University of Medical Sciences, Shiraz, Iran

**Keywords:** Cancer research, Immunology, Urology, Oncology, Bladder cancer, Lymph node, Survival Treg

## Abstract

**Introduction and methods:**

To clarify the role of CD4^+^ regulatory T cells in bladder cancer, we investigated the frequency of these cells in tumor draining lymph nodes of 50 patients with bladder cancer who underwent radical cystectomy using flow cytometry method. We also assessed their association with prognosis and survival.

**Results:**

On average, 30.13 ± 2.17% of lymphocytes in draining lymph nodes from patients with bladder cancer were positive for both CD4 and FOXP3 molecules. Analyses also showed that 9.92 ± 0.8% of CD4^+^ lymphocytes had a regulatory phenotype (CD4^+^CD25^+^FOXP3^+^CD127^low/neg^). The frequency of total CD4^+^FOXP3^+^ lymphocytes as well as regulatory T cells was significantly greater in patients with at least one tumor-involved lymph node compared to those with tumor-free nodes (P = 0.026 and P = 0.036, respectively). Mean FOXP3 expression in CD4^+^ lymphocytes was greater in patients with stage IV compared with those in stage III (P = 0.046). No other significant associations were found between the frequency of regulatory T cells and other clinicopathological characteristics or patient survival.

**Conclusions:**

The increased frequency of regulatory T cells in patients with involved lymph nodes suggests that these cells may negatively regulate antitumor immune responses in draining lymph nodes. Our findings may have implications for immunotherapy-based treatments for bladder cancer.

## Brief summary

Increased frequency of CD4^+^ regulatory T cells in draining-lymph nodes of bladder cancer patients suggests a negative role for these cells in antitumor immune responses.

## 1 Introduction

Bladder cancer (BC) is the sixth most common cancer among men worldwide [1]. Although the current histopathological classification has improved the clinical management of this disease, progression to invasive disease or recurrence are still significant challenges. One possible explanation for progression or recurrence is tumor evasion from antitumor immune responses, which is considered one of the hallmarks of cancer [2].

CD4+ regulatory T (Treg) cells that express the FOXP3 transcription factor are highly immunosuppressive cells with central roles in self-tolerance and immune homeostasis. These vitally important functions, however, coexist with detrimental effects on tumor immunosurveillance and antitumor immunity. Treg cells can diminish effective antitumor immunity through a variety of immunosuppressive mechanisms both in a contact-dependent manner and by secreting inhibitory cytokines; these effects collectively result in tumor progression [2]. Accordingly, the increased prevalence of Treg cells in the tumor microenvironment and circulation in patients with cancer, including urothelial malignancies, is commonly considered evidence of their involvement in disease pathogenesis and progression [3]. The infiltration of large numbers of Treg cells in tumors is also frequently associated with a poor prognosis and worse clinical outcomes in different types of cancer [4]. In BC as well as many other tumors, it has been shown that higher frequencies of these suppressive cells are correlated with a weaker response to therapy [5]. However, a positive correlation was also reported between FOXP3 expression in bladder tumor tissues and better patient survival [6].

Interpreting the results from different studies is made difficult by differences in sample type (tumor vs. blood), the limited numbers of cases, and the diversity of markers and analytical methods. To clarify the role of Treg cells in BC, the present study was designed to use flow cytometry to determine the frequency of different cells with a regulatory phenotype in tumor draining lymph nodes (TDLNs), which are the main site of antitumor immune responses. The data were analyzed to identify relationships between the frequency of different subsets and the main clinicopathological parameters of the disease as well as patient survival.

## 2 Results

### 2.1 Clinical and pathological characteristics of the patients

After confirmation by pathology, a total of 50 lymph nodes were obtained from patients with BC (mean age = 63.7 ± 12.4 years). According to the pathology reports, urothelial carcinoma was the most frequent tumor type (49/50, 98%). Twelve patients had at least one involved lymph node (LN^+^ patients; 24.5%). Most patients were in stage II (22/50, 44.9%) and 42 patients had tumors with a high histological grade (84%). The main clinicopathological characteristics of the patients are summarized in Table 1.

**Table 1:** Clinicopathological characteristics of patients with bladder cancer.

Characteristics	Value
Age (years)	63.7 ± 12.4
Gender	
Male	42 (84%)
Female	8 (16%)
**Survival**	**OS (months):** 21.87 (3.13–36.5)
Alive	21 (63.64%)
Dead	12 (36.36%)
Unreported	17
Tumor type	
Urothelial carcinoma (UC)	49 (98%)
Non-UC	1 (2%)
Histological grade	
Low grade	6 (12.5%)
High grade	42 (87.5%)
Unreported	2
Lymph node status	
Free (N0)	38 (76%)
Involved	12 (24%)
N1	4 (8.2%)
N2	7 (14.3%)
N3	1 (2%)
T grouping	
T1	5 (10%)
T2	30 (60%)
T3	7 (14%)
T4	8 (16%)
Stage	
I	5 (10%)
II	22 (44%)
III	9 (18%)
IV	14 (28%)
Muscular invasion	
Positive	45 (90%)
Negative	5 (10%)
Lymphovascular invasion	
Positive	18 (39%)
Negative	28 (61%)
Unreported	4
Perineural invasion	
Positive	27 (58.7%)
Negative	19 (41.3%)
Unreported	4
Urothelial CIS	
Positive	16 (43.2%)
Negative	21 (56.8%)
Unreported	13

All percentages are valid. Missing data were excluded from the calculations.

OS: overall survival; CIS: carcinoma in situ.

### 2.2 Phenotype determination of CD4^+^ subsets in TDLNs

**Figure 1:**
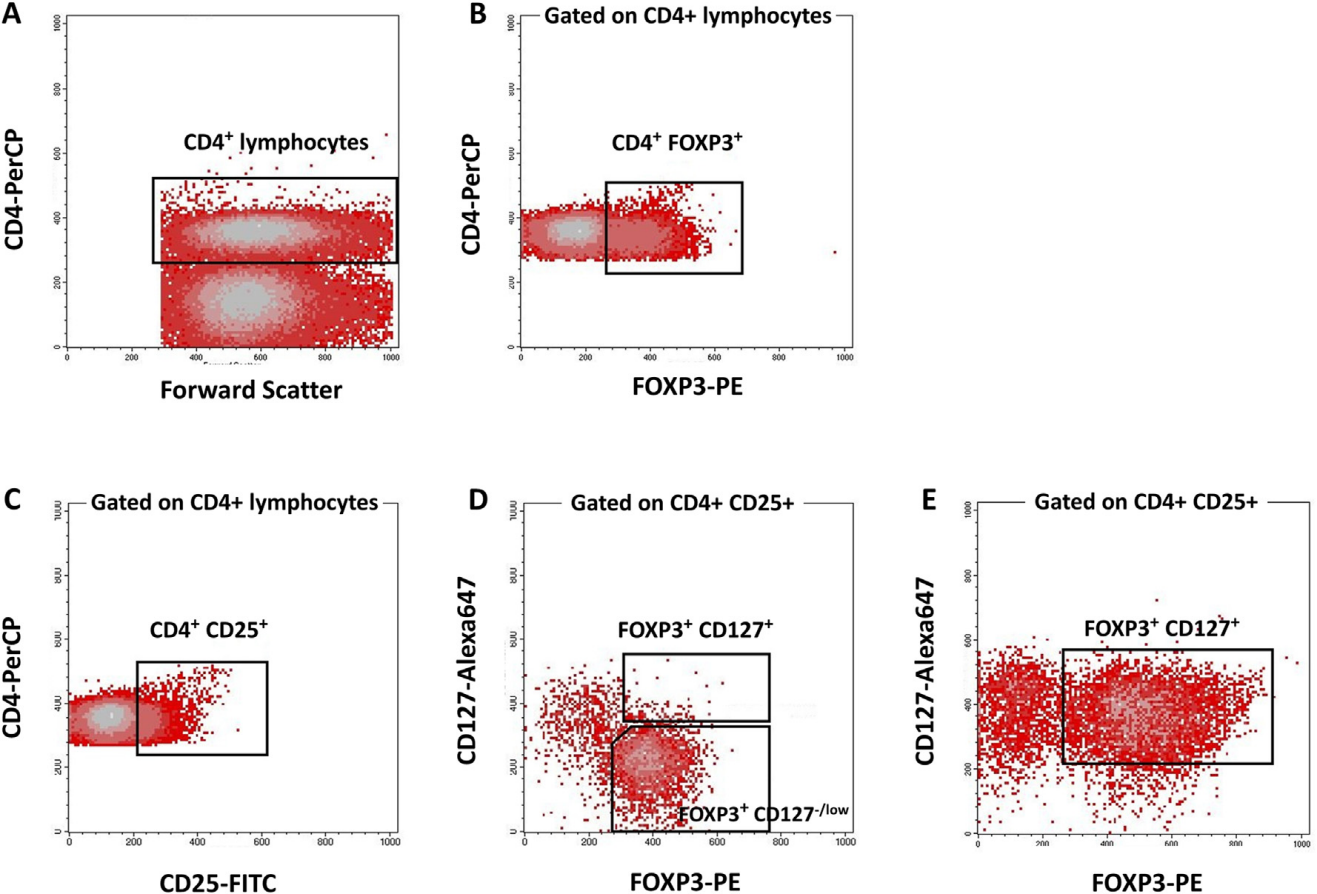
**Phenotype determination of different CD4**^**+**^**Treg subsets in tumor draining lymph nodes from patients with bladder cancer.** To determine the frequency of different CD4^+^ subsets, CD4^+^ cells were first selected in the lymphocyte gate (A). Beside determining total frequency of CD4^+^FOXP3^+^ cells (B), CD4^+^CD25^+^ lymphocytes (C) which were FOXP3^+^ with low or no expression of CD127 were considered regulatory T cells (D); CD4^+^CD25^+^FOXP3^+^ cells that were CD127^+^ were also assessed (D). In some cases, this phenotype was dominant among CD4^+^CD25^+^ lymphocytes (E).

To determine the frequency of different subsets of CD4^+^ Treg cells, after selecting CD4^+^ cells in the lymphocytes gate (Figure 1A), the phenotype of different FOXP3-expressing subsets was defined based on the expression of CD25, FOXP3 and CD127. CD4^+^CD25^+^FOXP3^+^ lymphocyte populations with low or no expression of CD127 (CD127^low/neg^) were considered Treg cells (Figure 1D); CD4^+^CD25^+^FOXP3^+^ cells that expressed CD127 were also assessed as CD127^+^ Treg cells (Figure 1E). For the CD4^+^ lymphocyte population, the average frequency of different CD4^+^ subsets along with the mean fluorescence intensity (MFI) of FOXP3 expression were recorded (Table 2).

**Table 2:** Frequency of different subtypes of CD4^+^ Treg cells in tumor draining lymph nodes from patients with bladder cancer.

Cell subset	Phenotype	Min	Max	Median	Mean ± SEM
CD4^+^FOXP3^+^	CD4^+^FOXP3^+^	10.26	69.19	26.03	30.13 ± 2.17
Treg	CD4^+^CD25^+^FOXP3^+^CD127^low/neg^	0.05	24.1	9.88	9.92 ± 0.8
CD127^+^ Treg	CD4^+^CD25^+^FOXP3^+^CD127^+^	0.28	16	2.79	3.74 ± 0.43
CD4^+^CD25^+^	CD4^+^CD25^+^	10.77	29.49	19.2	19.63 ± 0.74
CD4^+^CD127^+^	CD4^+^CD127^+^	51.29	99.7	75.93	75.08 ± 2.06
**Mean expression of FOXP3 molecule in different CD4+ subsets (based on MFI)**					
Treg	CD4^+^CD25^+^FOXP3^+^CD127^low/neg^	0.79	2.81	1.71	2.03 ± 0.22
CD127^+^ Treg	CD4^+^CD25^+^FOXP3^+^CD127^+^	1.19	4.6	1.55	1.86 ± 0.18
CD4^+^FOXP3^+^	CD4^+^FOXP3^+^	1.22	3.58	1.75	1.95 ± 0.12

MFI: mean fluorescence intensity.

### 2.3 Frequency of CD4^+^ Treg cells in TDLNs and different clinical and pathological parameters

**Figure 2:**
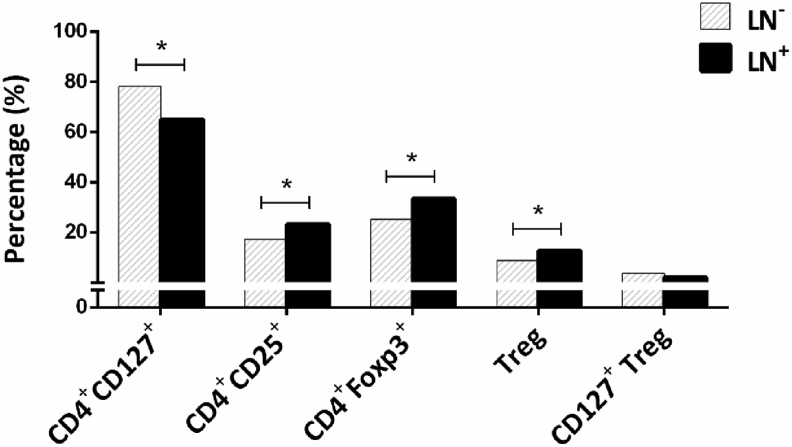
**Frequency of different CD4**^**+**^**lymphocytes in patients with different nodal status.** The data are presented as median values. ∗Significant difference at 0.05 (2-tailed test). LN^−^: Patients with tumor-free nodes. LN^+^: Patients with at least one tumor-involved node.

Our analysis indicated that 9.92% of CD4^+^ lymphocytes in TDLNs from patients with BC had a Treg phenotype (CD4^+^CD25^+^FOXP3^+^CD127^low/neg^). Comparisons of the percentage of different FOXP3-expressing CD4^+^ subsets in patients with different nodal status showed that the total frequency of FOXP3^+^CD4^+^ cells along with Treg cells was significantly higher in patients with at least one involved node (LN^+^ patients) compared to node-negative patients (LN^−^ patients) (P = 0.020 and P = 0.036, respectively; Figure 2). Moreover, the mean expression of FOXP3 (based on MFI) in CD4^+^FOXP3^+^ lymphocytes was also significantly greater in patients with stage IV disease compared to stage III (P = 0.046, without Bonferroni correction).

Association studies were also done between the percentage of CD4^+^ Treg subsets and other clinical and pathological characteristics, including T grouping, histological grade, muscular invasion, lymphovascular invasion, perineural invasion, and carcinoma in situ. Our analysis disclosed no significant differences among the percentages of different CD4^+^ subsets in patients with different clinical and pathological parameters; however, the percentage of the CD4^+^CD127^+^ population was significantly greater in T1 patients compared to T4 patients (P = 0.048, without Bonferroni correction).

Spearman correlation analyses were also done to determine the possible relationships between the prevalences of different subsets with each other, and with age. Although no correlations were observed between the frequency of different cell subsets and patients' age, there was a positive corelation between the frequencies of Treg cells and CD4^+^FOXP3^+^ cells (R = 0.323, P = 0.022). In contrast, negative corelations were seen between CD4^+^CD127^+^ and CD4^+^FOXP3^+^ cells (R = −0.342, P = 0.015) and between CD4^+^CD127^+^ cells and CD25^+^ Treg cells (R = −0.376, P = 0.007).

### 2.4 Survival analysis

We next assessed the association between the frequency of different Treg cell subsets in TDLNs and patients' survival. The mean follow-up period was 21.87 months (3.13–36.5 months). Among 33 patients with known survival status, 12 patients (36.4%) died. However, frequency analysis revealed no differences in the percentages of different Treg subsets or mean FOXP3 expression in draining lymph nodes between surviving and dead patients (P > 0.05).

Considering survival time, the univariate Cox regression model indicated that none of investigated subsets with a Treg phenotype affected survival. Nevertheless, pathological parameters including lymph node involvement (HR 3.768, 95% CI 1.047–13.559; P = 0.042) and organ-confined tumors (HR 5.254, 95% CI 1.355–20.375, P = 0.016) were significantly related with lower survival.

## 3 Discussion

We investigated the presence of CD4+ Treg cells in TDLNs from patients with BC. Interestingly, more than 30% of CD4+ lymphocytes in draining lymph nodes expressed FOXP3, considered a specified transcription factor for Treg cells. Our analysis of the association of these cells with the main clinicopathological parameters of the disease showed that the prevalence of FOXP3-expressing CD4^+^ lymphocytes was remarkably increased in patients with at least one positive node.

Based on our observation, the frequency of these FOXP3-expressing cells was much higher in draining lymph nodes from patients with BC compared to other cancers including breast and colon carcinomas [7, 8]. The difference may be due to both in situ generation at the tumor site and greater recruitment from the periphery in BC [9, 10]. It has been also reported that effector T cells can transiently express FOXP3 after activation. Although a direct association has been demonstrated between FOXP3 expression and suppressive activities of Treg cells, part of the FOXP3-expressing cells in patients with BC may be temporarily-activated T cells. Accordingly, in the next step we included other phenotypic markers for Treg cells: CD25 and CD127. Our data showed that approximately 10% of CD4+ lymphocytes in TDLNs of BC patients exhibited the CD4^+^CD25^+^FOXP3^+^CD127^low/neg^ phenotype. Along with FOXP3-expressing CD4+ lymphocytes, the prevalence of Treg cells was also significantly increased in patients with at least one positive node. Although no earlier studies of urological malignancies investigated Treg cells in draining lymph nodes from patients with BC, our study in patients with breast cancer, like the present study, found higher frequencies of Treg cells among LN^+^ patients as well as in those with more involved lymph nodes [7]. Similar increases in Treg cell populations have frequently been reported in other studies of tumor tissues [11], lymph nodes [7, 8] and peripheral blood [12, 13, 14] from patients with different types of cancer, including urological malignancies. Although the type of phenotyping assay differed between studies, elevated numbers of Treg cells in both tumor tissues and in the circulation in patients with BC were frequently reported to be associated with more aggressive phenotypes, i.e. more tumor growth, higher tumor grade, and invasion of or metastases to draining lymph nodes [15, 16]. The higher frequency of FOXP3^+^ cells and their higher CD3/CD8 ratio have been associated with poorer prognosis and shorter recurrence-free and overall survival, as well as with the response to systemic chemotherapy [5, 17, 18]. Most earlier studies also found that the number of Treg cells decreased notably after tumor removal [15, 16].

Consistent with the view that draining lymph nodes are the first line of defense against tumor dissemination, many studies have demonstrated that the structure and particularly the cellular composition of draining lymph nodes change dramatically during cancer progression and metastasis [19]. These changes usually provide suitable conditions for tumor cell metastasis, for example by increasing the frequency of pro-tumorigenic cells such as T and B cells with regulatory phenotypes, as well as favoring type 2 T cell responses as seen in patients with more aggressive tumors or by increasing in the expression of PD-1 immune checkpoint molecule [7, 8,[19], [20], [21], [22]]. The increased frequency of Treg cells in draining lymph nodes from patients with bladder tumors may therefore indicate that these cells, based on their nature and inhibitory functions, are key players in providing an immunosuppressive and pro-tumorigenic environment in BC which facilitates tumor cell metastasis and dissemination. This may also be a logical reason for high rate of recurrence observed in bladder tumors [9].

Although it has been reported that the level of CD127 expression is inversely correlated with FOXP3 expression and the suppressive function of CD4^+^ Treg cells [23], some studies found that during activation, Treg cells expressed higher levels of CD127 compared to nonimmunized settings, and that this expression was essential for enhanced survival and Treg-mediated suppression [24,25]. A population of memory Treg cells with high levels of CD127 expression has also been reported in mouse skin but not secondary lymphoid organs [26]. Although a similar phenotype for human memory Treg cells has not been found to date, in the present study we observed high expression of CD127 on CD4^+^CD25^+^FOXP3^+^ cells in many patients (Figure 1D). However, the frequency of these cells was not associated, in general, with difference in clinicopathological parameters. Whether these cells also have the properties of memory Treg cells or not remains to be elucidated.

We also investigated the association of different Treg subsets in TDLNs with patients' overall survival; however, we found no significant correlations. In this connection, Parodi et al. reported that the intratumoral ratio of effector to regulatory T cells in patients with recurrence was less than 1 [9]. It has also been shown that the frequency of Treg cells is an independent predictor of BC recurrence, and higher frequencies of Treg cells were associated with poor responses to treatment with intravesical Bacillus Calmette-Guerin as well as with shorter recurrence-free survival [17,27]. Consistent with these results, Jou et al. showed that FOXP3 knockdown inhibited tumor growth in a mouse model of BC, resulting in prolonged survival [10]. In addition, a retrospective study by Winerdal et al. found that the presence of FOXP3^+^ cells (more than 3 cells) in tumor infiltrating lymphocytes was associated with a better prognosis in non-muscle-invasive urothelial carcinoma, whereas the presence of FOXP3^+^ tumor cells showed an inverse correlation with survival [6]. It should be noted that different sites as well as small sample sizes in these studies limit the comparability of their results. In addition, as in the present study, no association was found between FOXP3 expression as a marker for Treg cells and clinical outcomes in other types of cancer (i.e. colorectal cancer) [28], whereas when different markers such as CD127, CTLA-4 and Blimp-1 were used alongside FOXP3 as Treg cell-defining markers, a positive association was observed with patient outcome [29,30]. Therefore, a subtype of Treg cells with a different phenotype may also have a significant effect on survival in patients with BC.

## 4 Conclusions

Taken together, our results show that the frequency of Treg cells was higher in patients with tumor-involved lymph nodes; however, no association was observed between Treg cell frequency and survival. Because several effector and memory subtypes of Treg cells have recently been characterized, using a comprehensive set of markers in larger samples of patients will be necessary to further clarify the role of Treg cells in the prognosis and survival of patients with bladder cancer.

## 5 Materials and methods

### 5.1 Patients

Fifty patients with BC who had undergone radical cystectomy and pelvic lymph node dissection were recruited for the study. After routine pathological examination, a portion of the dissected lymph nodes in culture medium was sent to the laboratory. Tumor infiltration in the nodes was determined histologically by expert pathologists. Clinical and pathological information was obtained from the patients' file. Written informed consent was obtained from all patients. The study was approved by the Ethical Committee of Shiraz University of Medical Sciences (IR.sums.med.ref.1396.S341).

### 5.2 Isolation of mononuclear cells from lymph nodes

To obtain single-cell suspensions, fresh lymph nodes were mechanically minced into small pieces in complete culture medium (RPMI 1640, Biosera, France) containing 10% fetal bovine serum (Gibco, USA) and 1% penicillin/streptomycin (Biosera) and filtered through a 40-μm cell strainer (BD Biosciences, USA). Mononuclear cells were then separated with Ficoll-Hypaque (Biosera) gradient centrifugation. The mononuclear ring was harvested, washed twice and dissolved in 1× phosphate buffered saline for further analysis. The cells were then counted with trypan blue dye (Biosera) to ensure viability.

### 5.3 Cell staining and flow cytometry analysis

To determine the frequency of T cells with a regulatory phenotype, mononuclear cells were first surface-stained with appropriate fluorochrome-conjugated antibodies for CD4 (PercP, clone: SK3; BioLegend, USA), CD25 (FITC, clone: M-A251; BD Biosciences) and CD127 (Alexa Flour®-647, clone: HIL-7R-M21; BD Biosciences) molecules. The cells were then fixed and permeabilized with FOXP3 Buffer Set according to the manufacturer's instructions (BD Biosciences), and were stained for intracellular FOXP3 (PE, clone: 259D/C7; BioLegend). At the same time, cells in a separate tube were used for surface and intracellular staining with appropriate isotype-matched controls. The cells were then washed, resuspended in PBS 1× and used for acquisition with four-color flow cytometry (FACSCalibur, BD Biosciences). At least 100 × 10^3^ events were read, and the data were analyzed with the CellQuest Pro software package. The frequencies of different subsets of FOXP3-expressing cells were counted in CD4^+^ populations.

### 5.4 Statistical analysis

All data were analyzed with SPSS (version 16, SPSS Inc., USA). Nonparametric Mann–Whitney U and Kruskal–Wallis H tests were used to compare the frequency of cells between two or more groups, respectively. The correlation between the prevalence of subsets and the patients' age was assessed with Spearman's rank correlation. P values less than 0.05 (two-tailed) were considered statistically significant. Graphs were generated with GraphPad Prism 6 software (GraphPad Software, San Diego, CA, USA).

## Declarations

### Author contribution statement

A. Ariafar: Conceived and designed the experiments; Contributed reagents, materials, analysis tools or data; Wrote the paper.

Y. Vahidi and M. Fakhimi: Performed the experiments; Analyzed and interpreted the data; Contributed reagents, materials, analysis tools or data; Wrote the paper.

N. Erfani: Conceived and designed the experiments.

A. Asadollahpour: Contributed reagents, materials, analysis tools or data.

Z. Faghih: Conceived and designed the experiments; Analyzed and interpreted the data; Contributed reagents, materials, analysis tools or data; Wrote the paper.

### Funding statement

This work was supported by 10.13039/501100004320Shiraz University of Medical Sciences, Shiraz, Iran (94-10835) and 10.13039/501100007432Shiraz Institute for Cancer Research (ICR-100-500).

### Declaration of interests statement

The authors declare no conflict of interest.

### Additional information

No additional information is available for this paper.
